# A core outcome set for acute otitis media (COS-AOM) for primary and community care studies

**DOI:** 10.1186/s12875-025-02821-1

**Published:** 2025-04-28

**Authors:** Esther T. van der Werf, Rachel Perry, Thomas Ostermann, Henrik Szőke, Alyson L. Huntley

**Affiliations:** 1 Homeopathy Research Institute (HRI), London, UK; 2 The National Institute for Health and Care Research Applied Research Collaboration West (NIHR ARC West) at University Hospitals Bristol and Weston NHS Foundation Trust, Bristol, UK; 3 Department for Psychology, Faculty of Health, University of Witten /Herdecke, Witten/Herdecke, Germany; 4 Department of Integrative Medicine, Faculty of Health Sciences (ETK), University of Pécs, Pécs, Hungary; 5 Centre for Academic Primary Care, Bristol Medical School, Bristol, UK

**Keywords:** Acute otitis media (AOM), Traditional complementary and integrative medicine (TCIM), Core outcome set (COS), Randomised controlled trial (RCT)

## Abstract

**Background and Objective:**

Outcome heterogeneity reported in Acute Otitis Media (AOM) research hinders evidence accumulation. Identification of a Core Outcome Set (COS) to report on in future studies in AOM is warranted.

**Methods:**

**Phase 1**: Candidate outcomes identification by reviewing previously reported outcomes in systematic reviews of AOM. **Phase 2**: In a Parent and Public Involvement (PPI) meeting candidate outcomes were discussed on their importance, presence, and absence. **Phase 3**: The clinical perspective of health professionals and pharmacists was anonymously gained through a ranking task. **Phase 4**: An International Steering Committee (ISC) discussed the ranked outcomes and advised on the final COS.

**Results:**

51 candidate outcomes were identified from 3 reviews and summarised to 20 overarching outcomes in Phase 1. Eight parents participated in the PPI meeting. 28 participants (11 GPs, 11 Traditional Complementary and Integrative medicine (TCIM) Professionals, 6 Pharmacists) ranked the 20 outcome cards. Moderate agreement in ranking was reached within all 3 medical groups, with pharmacists showing the highest agreement (0.540) and the TCIM professionals the lowest (0.421). Correlation coefficients between the groups show a sufficiently high agreement (*P* < 0.01). The ICS confirmed the final COS-AOM including 8 acute outcomes and 2 mid-long-term outcomes. Agreement for each outcome was reached with 100%.

**Conclusion:**

The proposed COS defines a minimum set of outcomes to be measured and reported in primary care and community studies on AOM, including TCIM clinical trials, to enhance evidence-based knowledge. Future research should focus on validating commonly used measurement tools for these outcomes and enhancing findings’ generalisability beyond the UK, Europe and primary care settings.

## Background

Approximately 80% of children will have at least one episode of Acute Otitis Media (AOM) [1, 2]. AOM is important to children, parents, the public, and the health care system: the infection causes pain and distress to the child and parents, frequently results in health service consultations and is the most common infection for which a child is given antibiotics worldwide [3–6].

While bacterial and/or viral pathogens can cause AOM, it is usually considered a bacterial complication of primarily viral upper respiratory tract infection [7]. National Institute for Health and Care Excellence (NICE) guidelines for AOM highlight that AOM is a self-limiting infection and that most children recover within 3 to 7 days without resorting to antibiotics. Serious complications are rare [8].

The inappropriate use of antibiotics is one of the key drivers of antibiotic resistance and is a global health priority [9]. With the rise in antibiotic overprescribing leading to antibiotic resistance, it is important to seek non-antibiotic prevention and treatment strategies. One of these strategies is using Traditional, Complementary and Integrative Medicine (TCIM) [10]. Previous studies have demonstrated the benefits of TCIM for symptom control [11, 12] and in reducing antibiotic prescriptions for respiratory tract infections in general [13] and antibiotic use [14–17].

However, the outcome measures and assessment protocols used to evaluate the effectiveness of AOM treatment in both conventional and TCIM studies are numerous and diverse. Trials evaluating interventions for AOM exhibit considerable heterogeneity in study design, interventions, populations, and outcome measures, hindering data meta-analysis and evidence accumulation. Therefore, identifying and validating a core outcome set (COS) to standardise outcomes reporting in randomised controlled trials (RCTs) is needed.

COS represent the minimum that should be measured and reported in all clinical trials of a specific condition [18]. These sets aim to create consensus on the outcome measures used in trials, thereby reducing the risk of outcome reporting bias and heterogeneity in outcome measurement [19]. This standardisation facilitates systematic reviews and meta-analyses, enhancing the interpretation of evidence to guide clinical practice. Initiatives like Core Outcome Measures in Effectiveness Trials (COMET) promote the standardisation of measurements across trials and maintain a database of COS [18].

Despite the increasing number of COS and the existence of a COS for OM with effusion [20, 21], none currently exist for conventional or TCIM research in AOM. This study aimed to develop a core outcome set for use in conventional- and TCIM trials assessing interventions for patients with AOM in primary care or community settings.

## Methods

A four-phase study was conducted to agree on a set of core outcomes. Due to the nature of the study, no ethical approval was requested.

### Phase 1: candidate outcomes

Candidate outcomes were identified by reviewing previously reported outcomes in systematic reviews of AOM. Using a selection of systematic reviews is an efficient, evidence-based, and methodologically rigorous approach to identifying candidate outcomes for a COS as it aims to leverage the most current and comprehensive evidence available. This allows the research team to build on pre-existing syntheses rather than duplicate efforts with new, broad scoping searches. It also aligns with the COMET guidelines [18], which emphasise the use of systematic reviews for COS determination.

We included a Cochrane Systematic Review (Gold standard of reviews) of AOM management [6], the most recently published (at the time of study) high-quality non-Cochrane Systematic Review of AOM [22], and a systematic review of TCIM and (A)OM (completed at the time of the study) [23]. As the last study included both AOM and Otitis Media with Effusion (OME), only the studies reporting AOM outcomes were included as candidate outcomes. All outcome measures of the studies included in the systematic reviews were used to identify the initial list of unique candidate outcomes for the COS.

### Phase 2: parent and public involvement

Phase two involved Parent and Public Involvement (PPI). The opinions of parents on the treatment of AOM in children are important because it is this group that most closely observes the benefits and adverse effects of treatments given to their children. The outcomes identified by parents and carers are likely to be grounded in their accounts of what matters to them about AOM and its treatment, which is in keeping with the principle of privileging the lay perspective [24]. Through an existing University of Bristol PPI group, we identified eight parents/carers (with lived experience) to participate in a 1.5-hour PPI meeting. Before the meeting, the participants were e-mailed information, including a brief plain English description of a COS and the purpose of the meeting. Using an adapted form of the nominal group technique, we asked our PPI members to discuss the candidate outcomes [25]. Each participant was allowed to discuss the candidate outcomes and give their view on their importance, presence, and absence. If the discussion identified any new outcomes, they were also considered for inclusion in phase 3. After structured small-group discussions led by the researchers, the final list of candidate outcomes for use in Phase 3 was confirmed.

### Phase 3: clinical perspective

Phase three involved gaining the clinical perspectives of GPs, TCIM professionals and Pharmacists. Optimal Workshop Card Sort was used to anonymously collect ratings for the candidate items (defined in phase 2) for the COS. Participants were asked to drag the outcomes from the left side of the webpage to cards named Priority Outcome 1 to Priority Outcome 20 on the right side of the webpage. When no outcomes remained on the left side, they were asked to complete the task and close the webpage. The task was estimated to take a maximum of 10 min, based on piloting among the research team.

We used overarching organisational network emails and newsletters to invite GPs, TCIM professionals and Pharmacists to participate. The network email and newsletter text included background information on the study, the need for a COS, and a link to the ranking exercise. It also confirmed that no personal data or practice information was being gathered. Table 1 presents the networks and organisations used for invitation dissemination. Further dissemination among invites’ networks was encouraged. We aimed for a minimum of 10 responses from each medical group based on previous COS studies [26].

**Table 1:** Dissemination of invitation, including study information and link to the ranking exercise

Participants	Network/Organisation	Website
GPs	Primary Care Academic CollaboraTive (PACT) network, University of Bristol, UK Centre for Academic Primary Care (CAPC), University of Bristol	www.gppact.org www.bristol.ac.uk/primaryhealthcare
TCIM professionals	National Centre for Integrative Medicine (NCIM)	www.ncim.org.uk
Pharmacists	Southwest Pharmacy Research Network	www.bath.ac.uk/projects/south-west-pharmacy-research-network
GPs TCIM professionals Pharmacists	Global Initiative for Traditional Solutions to Antimicrobial Resistance (GIFTS-AMR)	www.jpiamr.eu/projects/gifts-amr

### Statistical analysis

To analyse the card-sorting task, Kendall’s coefficient of concordance W [27] was calculated based on the ranks given for the 20 outcomes (total group and subgroups). Kendall’s W ranges from 0 (no agreement) to 1 (complete agreement). According to Landis [28], the following categories were defined: 0.00 < = W < 0.20 - Slight agreement; 0.20 < = W < 0.40 - Fair agreement; 0.40 < = W < 0.60 - Moderate agreement; 0.60 < = W < 0.80 - Substantial agreement; 0.80 < = W < 1.00 - Almost perfect agreement). The mean ranks for each outcome were analysed in a correlation and regression analysis to compare the three subgroups further.

### Phase 4: international steering committee

The results of the card-sorting task were presented to the International Steering Committee (ISC). Participants were sampled to achieve an international balanced representation of patients (Phase 1 PPI member), healthcare professionals and researchers (trialist and statistician). The goal of the ISC meeting was to decide which outcomes would be included in the final core outcome set. This meeting was chaired by an independent researcher with expertise in consensus methodology, who was not a research team member. We aimed to have a small representative group to enable meaningful small-group discussions and international participation to ensure the COS was broadly accepted, which was possible because of online participation.

The format of the ISC meeting comprised a short overview of the study and a summary of the results of the card-sorting task by the three medical groups. The ranked order of the 20 outcomes and the correlations between the mean ranks were presented. After discussing the separate outcomes, ISC members were asked if there were any fundamental reasons why some outcomes should not be included in the COS. Divergent views were actively sought, and the chair ensured everyone had the opportunity to participate in discussions. In case of disagreement, voting (putting hands up) was used. The consensus criterion was set at 70% for inclusion [29, 30]. Outcomes meeting the criteria for consensus were included in the COS; all other items were excluded. The pre-final COS was discussed to determine coherence between the outcomes, hence having fewer, broader outcomes. The meeting finished with a summary of the discussion. After the meeting, the final COS was sent to all ISC members for confirmation.

## Results

**Fig. 1:**
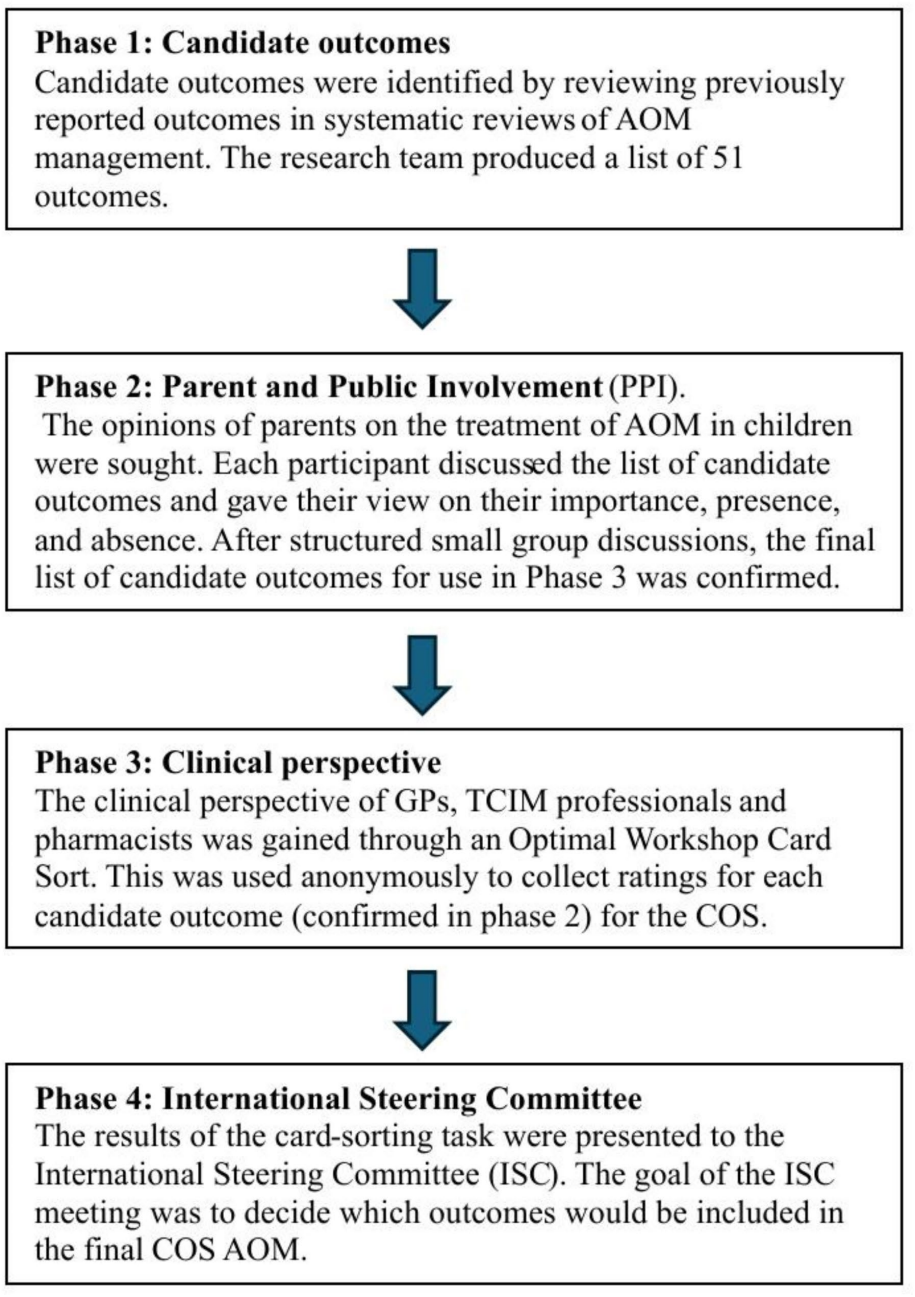
Summary of the COS-AOM process

Figure 1. summarises the 4 phases of the COS-AOM process (Fig. 1).

### Phase 1: candidate outcomes

51 Candidate outcomes were identified from the three systematic reviews of AOM management (attachment 1). After deduplicating and combining similar candidate outcomes, the candidate outcomes were categorised under the following domains: Pain (*n* = 3), Symptom Relief (*n* = 4), Physical Measures (*n* = 3), Treatment failure (*n* = 3), Antibiotic use (*n* = 2), Adverse events (*n* = 1), Microbiology (*n* = 1), Health care use (*n* = 1), Impact on family (*n* = 2) (attachment 2). The candidate COS for PPI input included 20 outcomes.

### Phase 2: parent and public involvement (PPI)

Eight parents/carers (with lived experience (of a child (with AOM) responded to the request for participation in the 1.5-hour lasting PPI meeting. Based on the general discussion and the structured small group discussions in which the participants discussed the 20 outcomes, “Sleep disturbance” was added to the outcome set for Phase 3. The parents believed this outcome was particularly important and should be a separate outcome rather than an example of the outcome “Symptom relief”. To keep the total outcomes to 20, the outcomes “Presence of middle ear effusion (MEE)” and “Time to resolution of MEE” were advised to be combined for the next phase by the PPI members.

### Phase 3: clinical perspective

In total, 28 participants completed the sorting task and ranked the symptoms from 1 to 20 in priority (Attachment 3). Of those, 11 participants were GPs, 11 were TCIM professionals, and 6 were Pharmacists. Despite maximum efforts, the number of pharmacists did not reach the intended number of at least 10.

Kendall’s W in all subgroups reveals a moderate agreement among the raters, with the pharmacists showing the highest agreement of 0.540 and the TCIM professionals showing the lowest (0.421). For the total group (*n* = 28), however, the value of Kendall’s W is noticeably lower, which indicates a discordance between the groups regarding the ranking of the symptoms (Table 2). Thus, a pairwise comparison of the mean ranks is performed (Table 3).

**Table 2:** Agreement between ranking groups

	*N*	Kendall’s W	χ^2^	*p*-value
GPs TCIM Professionals Pharmacists **Total**	11 11 6 **28**	0.503 0.421 0.540 **0.426**	105.125 88.075 61.552 **226.492**	< 0.001 < 0.001 < 0.001 **< 0.001**

*0.00 < = W < 0.20 - Slight agreement; 0.20 < = W < 0.40 - Fair agreement; 0.40 < = W < 0.60 - Moderate agreement; 0.60 < = W < 0.80 - Substantial agreement; 0.80 < = W < 1.00 - Almost perfect agreement [28]

**Table 3:** Mean ranking for outcomes 1–20

Symptom	GPs	TCIM Professionals	Pharmacists	Total
Symptom Severity Pain Severity Pain Duration Symptom Duration Fever > 38 degrees C Recurrence of ear infection Use of Pain Medication Antibiotic use/absence of antibiotic use Presence of middle ear effusion (MEE) Recovery Number of recurrences in next 12 months Unscheduled medical visits/hospitalisation Sleep disturbance Time to initiate antibiotic treatment Days of work/ school missed Parent satisfaction with ear infection care Audiometry results (hearing problems) Glue ear/ear fluids Adverse events to antibiotics New related infections	3.09 (1) 4.09 (2.5) 4.09 (2.5) 4.91 (4) 6.73 (5) 10.73 (11) 10.64 (10) 8.73 (6) 12.00 (12) 9.55 (7) 12.45 (14) 10.27 (9) 9.91 (8) 14.18 (16.5) 12.27 (13) 15.18 (18) 14.18 (16.5) 17.36 (20) 13.27 (15) 16.36 (19)	3.09 (1) 5.18 (4) 4.36 (3) 4.18 (2) 9.64 (8) 10.18 (9) 9.36 (6.5) 10.73 (10) 9.27 (5) 11.64 (13) 9.36 (6.5) 11.27 (11) 14.91 (17.5) 12.55 (14) 14.91 (17.5) 11.55 (12) 15.00 (19) 13.45 (16) 16.18 (20) 13.18 (15)	2.83 (1) 3.33 (2) 5.17 (4) 4.67 (3) 6.67 (6) 6.33 (5) 10.00 (8) 11.67 (10.5) 9.00 (7) 11.67 (10.5) 11.00 (9) 13.83 (16) 12.50 (14) 12.33 (12.5) 14.67 (17) 16.50 (19) 12.83 (15) 12.33 (12.5) 16.00 (18) 16.67 (20)	**3.04** (1) **4.36** (2) **4.43** (3) **4.57** (4) **7.86** (5) **9.57** (6) **10.00** (7) **10.14** (8) **10.29** (9) **10.82** (10) **10.93** (11) **11.43** (12) **12.43** (13) **13.14** (14) **13.82** (15) **14.04** (16) **14.21** (17) **14.75** (18) **15.00** (19) **15.18** (20)

The ranking deviates for the symptoms “Recurrence of ear infection,” “Presence of middle ear effusion (MEE),” and “Parent satisfaction with ear infection care.” Nevertheless, Pearson’s correlation coefficients between the mean ranks of the respective groups, shown in Table 4, show a sufficiently high agreement between the groups of raters. The proportion of unexplained variance in the three medical professions, with values between 24% and 35%, is notably low.

**Table 4:** Correlations among the mean rankings between the three medical groups

	GPs	TCIM Professionals	Pharmacists
GPs		.692*	.841*
TCIM Professionals	.806*		.870*
Pharmacists	.764*	.856*	

*Pearson correlations* (*significant: *p* < 0.01)

### Phase 4: international steering committee

An International Steering Committee (ISC) with members from three countries (United Kingdom, Germany, and Switzerland), including researchers, clinicians, and methodologists, worked on developing the COS. The ISC reduced the number of outcomes from 20 to 10. Table 5 shows the committee members’ agreement on whether the outcomes should be removed (*n* = 8), combined (*n* = 2), or changed (*n* = 2). Agreement for each outcome was reached with 100%. Based on the advice of the ISC, we removed the health economic evaluation outcomes (“Unscheduled medical visits/hospitalisation” and “Days of work/ school missed”) from the COS due to the incompleteness of the health economic evaluation outcomes listed in the original 20 outcomes. Including cost-effectiveness in an RCT is warranted, but it will depend on the research question, the research setting, and available funding. Therefore, the ISC advised to refer to accepted guidelines on cost-effectiveness analysis for healthcare settings instead, for example, The Consolidated Health Economic Evaluation Reporting Standards (CHEERS) statement [31] or The guidelines for economic evaluation in healthcare (Version 2024) [32].

**Table 5:** International steering committee agreement on outcomes for final COS

		Agreement Experts (> 70%)			
**Outcome** (ranking based on Phase3)		**Keep**	**Remove**	**Combine**	**Change name/ranking**
1	Symptom Severity	Y			
2	Pain Severity	Y			
3	Pain Duration	Y			
4	Symptom Duration	Y			
5	Fever > 38 degrees C	Y			
6	Recurrence of ear infection	Y			
7	Use of Pain Medication	Y			
8	Antibiotic use/absence of antibiotic use	Y			
9	Presence of middle ear effusion (MEE)	Y		Outcome 9 and 18	Mid-long term outcome
10	Recovery	N	key elements of recovery included in outcomes related to pain, symptoms and MEE		
11	Number of recurrences	Y			Mid-long term outcome
12	Unscheduled medical visits/hospitalisation	N	Economic evaluation outcome: not part of the final COS (see results)		
13	Sleep disturbance	N	Included in outcome related to symptoms		
14	Time to initiate antibiotic treatment	N	Not relevant		
15	Days of work/ school missed	N	Economic evaluation outcome: not part of the final COS (see results)		
16	Parent satisfaction with ear infection care	N	Not relevant		
17	Audiometry results	N	Not measured in daily clinical (GP/Community) practice		
18	Glue/ear fluids	Y		Outcome 9 and 18	
19	Adverse events to antibiotics	Y			Adverse events/Serious complications
20	New related infections	N	Not relevant		

Finally, the ISC advised on differentiating between acute and mid-long-term outcomes. These headings have been added to the final COS, and the outcomes have been reorganised, considering the priority ranking (Table 3). Table 6 presents the final ten outcomes of the COS- AOM for future conventional and TCIM studies in a primary care/community setting. All ISC members have confirmed the final COS-AOM.

**Table 6:** Final COS acute otitis media to be used in future studies in primary care/community setting (in order of ranked importance)

Acute Outcomes	
1	Symptom severity*
2	Pain severity
3	Pain duration
4	Symptom duration
5	Fever > 38 Degrees C
6	Pain medication use
7	Antibiotic use
8	Adverse events/Serious complications
**Mid-Long term outcomes**	
9	Recurrence/number of recurrences in the next 12 months
10	Presence of middle ear effusion (MEE)/Glue ear/ear fluids

**e.g. fever, irritability, unusual crying or screaming, lack of drive, loss of appetite, unusual sleep behaviour

### Outcome measurement instrument

An outcome measurement instrument refers to how the outcome is being measured. This can be, among others, a single question, a questionnaire, a score obtained through physical examination, a laboratory measurement, or a score obtained through observation of an image [33]. Recommendations on the best tool to measure the outcomes “Symptom Severity” and “Pain Severity” in patients with AOM is currently lacking. Examples of measurement tools used in previous clinical trials in AOM for Symptom Severity are: The ear treatment group symptom questionnaire, 5 items questionnaire (ETG − 5) [34], Severity of Symptoms Scale (AOM-SOS) [35], AOM-Faces Scale (AOM-FS) [35], Functional status based on FS II R scores [36] and Symptom scores from parental diaries. Pain Severity is most commonly measured using a pain rating scale for pain intensity to assess the presence/absence of typical clinical symptoms, the Sum-of-pain-intensity differences (SPID) [37] or a parent-completed assessment of pain.

A severity scoring system that assigns a diversified score based on the following parameters: child’s age, intensity of otalgia, level of fever, intensity of crying/irritability, degree of Tympanic Membrane (TM) hyperemia, presence of TM bulging, presence of otorrhea [38] may cover the most important symptoms and signs from a patient’s and clinicians’ perspective. Review and validation of the sensitivity and specificity of the commonly used measurement tools for “Symptom Severity” and “Pain Severity” in patients with AOM is warranted to make recommendations for use in future studies in AOM management.

## Discussion

The COS-AOM study presents the development of the COS-AOM and the steps involved in reaching consensus. Our COS-AOM for future primary or community care studies, including TCIM settings, includes the acute outcomes: symptom severity and duration, pain severity and duration, fever, pain medication and antibiotic use and adverse events. In addition to these eight acute outcomes, two mid to long-term outcomes have been defined: recurrence and MEE/glue/ear fluids. The unanimous acceptance of the presented outcomes means we can be assured that the COS is widely accepted. The development of the COS-AOM is essential for ensuring that future research reporting is comprehensive, includes the patient’s perspective, and allows for study comparison and meta-analysis.

To further standardise outcome data collection and analysis, we recommend validation of the most commonly used tools for measuring these core outcomes in future studies. This is particularly important given that lack of agreement on up-to-date measurement tools has been identified as a barrier to COS uptake [39]; promoting uptake of our COSs by researchers and policy decision-makers is a critical next step.

The COS-OM study has several strengths. The methods were guided by established COS methodology, and active PPI- and ISC involvement ensured meaningful input and relevance from the patient’s, clinical and methodologist’s perspective. Including conventional GPs and TCIM professionals in our consensus process allows a broad uptake for future study designs testing non-antibiotic prevention and treatment strategies for patients with AOM in primary and community care. The study is strengthened by including the pharmacist’s perspective in the COS-AOM development. This is because the community pharmacists’ role in treating common minor ailments, such as coughs, colds, sore throats and ear infections, is becoming more acknowledged [40]. For example, the UK “Pharmacy First” initiative (www.england.nhs.uk/primary-care/pharmacy/pharmacy-services/pharmacy-first) enables community pharmacists to supply prescription-only medicines, including antibiotics and antivirals, where clinically appropriate, and ear infection is one of the seven common health conditions that fall under this initiative and can be treated without visiting a GP. Methodological advice from a researcher’s perspective (trialist and statistician) confirmed adaptability for future studies, including RCTs and Real-World Evidence (RWE) studies.

However, there were also limitations. While it is justified that the selection of candidate outcome measures was based on a chosen sample of systematic reviews [20], this approach, which strategically selected focused evidence from existing high-quality sources, might be considered a limitation. The key reasons for this selected approach were: (1) The COMET Initiative recommend systematic reviews as a key tool for identifying outcomes for inclusion in a COS [18], (2) Conducting a scoping review to identify both systematic reviews and primary research is a time-consuming process. Not only does it involve searching for and including primary studies, but it also requires mapping and synthesizing a broad range of outcomes, many of which may have already been comprehensively analysed in existing systematic reviews and (3) By selecting recent and relevant systematic reviews that have already identified, evaluated, and synthesized the primary studies makes the process of determining outcomes for the COS more efficient as it allowed us to focus on the synthesis and validation of candidate outcomes rather than conducting a new, exhaustive search and compiling data extraction. As AOM is a very discreet topic area, we are confident that these robust, on-target systematic reviews provided us with a comprehensive baseline of symptoms.

Given the central role of regulators and funders for trial endpoints considered in policy decision-making, the COS might have benefitted from the perspectives of a policy advisor representing a vital group. Although one of the medical experts of the ISC regularly advises on strategies and policies for infectious diseases and TCIM, the policy advisor’s perspective could be considered for additional feedback. The ISC consensus process included international panellists, which can give our findings greater generalisability. This COS is relevant to English-speaking countries, including the US and parts of Europe, but with the acknowledgement that there will be country-specific variations. Due to worldwide differences in and access to (primary care) health systems, the COS-AOM might need to be adapted for country-specific settings. Future research can expand the international representation to improve the generalisability of the outcomes beyond the UK/Europe and the primary care context. Although the COS-AOM is aimed at research in both the pediatric and adult populations, the emphasis in the PPI and ISC discussions has been on its use for future pediatric research, as AOM is more common in children than in adults.

Harmonised outcome measurement across studies and research settings is feasible for standard clinical measurements (e.g. fever) and self-completed questionnaires (e.g. symptom- and pain severity), but it may be challenging to objectively measure the mid-long term outcome presence of MEE, which require specific qualifications/tools which may not be available in a primary care or community setting in all countries. Although we acknowledge that resource constraints may be a barrier to measuring some outcomes, our rigorous multi-perspective consensus approach underscores the importance of the selected outcomes for determining the effectiveness of an intervention for symptom control and reducing antibiotic use in patients presenting with AOM symptoms to primary or community care.

## Conclusions

The COS-AOM study presents the first COS-AOM for primary and community care. Implementation of the COS-AOM in future studies will help to make the best use of limited research resources due to consensus-based outcome selection and reporting, facilitating comparison and synthesis across studies. By including both conventional and TCIM professionals, we aimed to fill a critical research gap, ensuring that future studies in TCIM also incorporate significant endpoints.

## Data Availability

All data generated or analysed during this study are included in this published article and its supplementary information files.

## Abbreviations

AOM: Acute Otitis Media
COS: Core Outcome Set
GP: General Practitioner
ICS: International Steering Committee
MEE: Middle Ear Effusion
PPI: Parent and Public Involvement
TCIM: Traditional Complementary and Integrative Medicine
RCT: Randomised Controlled Trial
